# Molecular basis of hypohidrotic ectodermal dysplasia: an update

**DOI:** 10.1007/s13353-015-0307-4

**Published:** 2015-08-21

**Authors:** Wieslaw H. Trzeciak, Ryszard Koczorowski

**Affiliations:** Department of Biochemistry and Molecular Biology, Poznan University of Medical Sciences, 6, Swiecickiego St., 60-781 Poznan, Poland; Department of Gerostomatology, Poznan University of Medical Sciences, 6, Swiecickiego St., 60-781 Poznan, Poland

**Keywords:** Differentiation of skin appendages, Hypohidrotic ectodermal dysplasia, TNFα-related signaling pathway

## Abstract

Recent advances in understanding the molecular events underlying hypohidrotic ectodermal dysplasia (HED) caused by mutations of the genes encoding proteins of the tumor necrosis factor α (TNFα)-related signaling pathway have been presented. These proteins are involved in signal transduction from ectoderm to mesenchyme during development of the fetus and are indispensable for the differentiation of ectoderm-derived structures such as eccrine sweat glands, teeth, hair, skin, and/or nails. Novel data were reviewed and discussed on the structure and functions of the components of TNFα-related signaling pathway, the consequences of mutations of the genes encoding these proteins, and the prospect for further investigations, which might elucidate the origin of HED.

## Introduction

Ectodermal dysplasias comprise a large group of congenital developmental disorders of at least two ectoderm-derived structures: eccrine sweat glands, teeth, hair, skin, and/or nails. Out of over 170 types of dysplasia (Pinheiro and Freire-Maia 1994; Priolo and Lagana 2001) the most common is ectodermal dysplasia 1 (OMIM 305100), an X-linked hypohidrotic form (HED), which occurs in the general population with a frequency of one per 17,000 live births.

HED embraces a genetically heterogeneous group of diseases and is due to mutations of several genes that encode components of the tumor necrosis factor α (TNFα)-related signaling pathway (Cluzeau et al. 2011). Mutations of these genes disturb the interaction, which takes place during embryonic development, between surface-located epithelial cells and the underlying mesenchyme (Schmidt-Ullrich and Paus 2005). In consequence, the process of initiation, formation, and differentiation of skin appendages is disturbed.

It has been demonstrated that HED is caused by mutations of several genes: *ED1*, encoding a ligand-ectodysplasinA-A1 (EDA-A1), *EDAR*, coding for ectodysplasinA-A1 receptor, *EDARADD*, programming the structure of EDAR-associated death domain protein and *NEMO* whose protein product, NFκB essential modulator (NEMO), is necessary for an indirect activation of nuclear factor κB (NFκB). With the exception of *EDA1* and *NEMO*, both localized on the X chromosome, all other genes encoding components of the TNFα-related signaling pathway involved in differentiation of skin appendages, are localized on the autosomes (Table 1).

**Table 1:** Mutation of genes encoding proteins involved in TNFα-related signal transduction pathway

Protein	Gene	Cytogenetic location	OMIM number*	Main types of mutation*							Total
				Missense/nonsense	Splicing defect	Small deletion	Small insertion	Small in/del	Gross deletion	Gross in/del	
Ectodysplasin A1	*EDA*	Xq12-q13.1	305100	117	20	33	12	2	21	1	206
Ectodysplasin A1 receptor	*EDA1R* (*EDAR*)	2q 12.3	129490d 224900r	25	5	6	2	1	2	--	41
Ectodysplasin A2 receptor (XEDAR)	*EDA2R* (*XEDAR*)	Xq12	-	-	--	--	--	--	--	1	1
EDAR-associated death domain	*EDARADD*	1q43	606603	5	--	1	--	--	--	--	6
NF-κB essential modulator	*NEMO*	Xq28	300291	44	6	21	15	--	2	2	90
TNF receptor-associated factor 6	*TRAF6*	11p12	-	--	--	1	--	--	--	--	1

*OMIM number designates disease **In/del, insertions/deletions; d, dominant; r, recessive

Within the last 10 years significant progress has been made in understanding the pathogenesis of HED and this is mainly due to the discovery of unknown proteins and the elucidation of their function in signal transduction via the TNFα-related pathway.

The purpose of this report is to review current literature on the structure and function of components of the TNFα-related signaling pathway, to present a novel approach to their contribution in the differentiation of skin appendages and to discuss the role of mutations of genes encoding components of this pathway in the origin of HED.

Mutations of these genes are responsible for systemic tooth agenesis, in addition to the defects of other ectodermal structures, and this review is limited to description of the function of their protein products.

The molecular basis of non-syndromic tooth agenesis, which is caused by mutations of other genes mainly *WNT10A*, (Arte et al. 2013; He et al. 2013; van den Boogaard et al. 2012) but also *MSX1* (Wang et al. 2011) and *PAX*9, ( Paixao-Cortes et al. 2011) will not be discussed, because it was thoroughly reviewed by several authors (Kapadia et al. 2007; Mostowska et al. 2003; Yin and Bian 2015).

## Signal transduction from ectoderm to mesenchyme through the TNFα-related pathway

The signaling via the TNFα-related pathway, which is critical for differentiation of skin appendages, has been elucidated mainly due to the investigations performed in mice (Mikkola 2009). It is initiated by one of the Wnt (wingless-type MMTV integration site) family of ligands (Rao and Kuhl 2010) probably WNT10A (Adaimy et al. 2007; Nawaz et al. 2009), which activates receptors belonging to the frizzled (Dann et al. 2001) and LRP (LDL receptor-related protein) (May et al. 2007) families. The downstream signal transduction toward Eda-A1 involves the canonical Wnt/β-catenin pathway (Clevers 2006) and requires lymphoid enhancer-binding factor-1 (Lef-1) for activation of the *Ed1* (*Ta*) expression (Durmowicz et al. 2002). In addition to its role in regulating *Ed1* expression, the Wnt/β-catenin pathway also regulates expression of *Edar* (Laurikkala et al. 2002).

Murine Eda-A1 (Srivastava et al. 1997), encoded by the *Ta* (*Tabby*) gene (Srivastava et al. 1997), contains 391 amino acids and its two amino acids shorter isoform, Eda-A2 encoded by the same gene (Yan et al. 2000) belong to the TNFα family of ligands and are involved in epithelial-mesenchymal interactions (Ezer et al. 1999; Mikkola and Thesleff 2003). They are transmembrane type II proteins with the C-terminus projecting outward (Ferguson et al. 1997; Kere et al. 1996; Monreal et al. 1998). Human EDA-A1 and EDA-A2 contain the same number of amino acids as their murine orthologues, and their structure is highly homologous to murine proteins. The C-terminal sequence of EDA-A1 and EDA-A2 (last 62 or 60 amino acids, respectively) is highly homologous to the C-terminal sequence of TNFα receptor ligands and forms a triple helix with the same sequences of two other identical molecules. The trimeric EDA-A1 (or EDA-A2) are stabilized by disulfide bonds formed by cysteine residues (Ezer et al. 1999).

The triple helix can be formed because the C-terminal domain (130 or 128 amino acids respectively) contains two clusters of collagen-like Gly-X-Y repeats (4 and 23 respectively). Near the shorter cluster there is an amino acid sequence: Arg-Val-Arg-Arg156-Asn-Lys-Arg representing overlapping consensus cleavage sites (Arg-X-Lys/Arg-Arg) recognized by furin-related protease that converts the C-terminal domain into a free ligand (Elomaa et al. 2001). Missense mutations, which occur most frequently at Arg156, account for approximately 20 % of HED cases (Chen et al. 2001; Elomaa et al. 2001).

The binding sites for the two ligands are located in the N-terminal domains of different receptors. The Edar1(Edar) binds only Eda-A1 while another receptor, Edar2 (named Xedar), binds exclusively Eda-A2 (Bayes et al. 1998; Yan et al. 2000).

Both Edar and Xedar are transmembrane proteins composed of an N-terminal, extracellular domain, a single transmembrane domain, and a C-terminal, intracellular region (Headon and Overbeek 1999).

Murine Edar, encoded by the *dl* (*downless*) gene, and its human orthologue, EDAR, encoded by the *EDAR* gene, belong to the family of the TNF receptors. Human EDAR contains 448 amino acids and exhibits 91 % homology to its murine equivalent (Headon and Overbeek 1999; Koppinen et al. 2001; Monreal et al. 1998). Both murine and human receptors comprise regions highly homologous to the TNF receptors, accounting for trimerization and ligand binding, and contain the death domain (Headon et al. 2001; Koppinen et al. 2001; Yan et al. 2000). Within the death domain there is a region interacting with proteins TRAF6 and TRAF3 (Naito et al. 2002), which transfer the signal down the pathway.

The structures of murine Xedar and its human orthologue XEDAR are similar but much smaller (both receptors contain only 297 amino acids each). Like Edar and EDAR, they comprise sequences highly homologous to the TNF receptors, and both contain the death domain. These sequences are responsible for trimerization and ligand binding.

Eda-A1 binding to Edar leads to the recruitment of a protein named Edaradd (*Edar associated death domain*), which binds to the C-terminal region of Edar (Chen et al. 2001; Elomaa et al. 2001).

Murine Edaradd, encoded by the *cr* (*crinkled*) gene and its human orthologue EDARADD, encoded by *EDARADD*, show 80 % homology. Both proteins contain 198 amino acids and comprise a structural motif, similar to that present in Myd88, which participates in signal transduction involving interleukin 1 and Toll receptors (Yan et al. 2002). This motif is responsible for binding to the death domain of Edar or EDAR (Headon et al. 2001; Yan et al. 2002).

Edaradd is co-expressed with Edar in epithelial cells during the formation of skin appendages. It acts as an Edar adaptor molecule linking this receptor to a RING-domain of Traf6 protein (Megas et al. 2011), which strongly activates the NFκB (nuclear factor κB) pathway (Headon et al. 2001; Yan et al. 2002), and weakly activates the JNK pathway as well as the caspase-independent cell death pathway (Kumar et al. 2001; Mikkola and Thesleff 2003).

In contrast, the binding of Eda-A2 to Xedar leads to an interaction of its intracellular domain with Traf3 and/or Traf6, and in the consequence activation of both JNK (c-Jun N-terminal kinase) and NFκB pathways (Sinha et al. 2002). This interaction, however, does not require Edaradd. In humans, these relations seems identical.

## Activation of the NFκB pathway

The NFκB pathway is activated by TRAF proteins TRAF6 being the principal one (Naito et al. 2002). TRAF6, is an E2 ubiquitin (E2) ligase and acts as a homodimer, which interacts with the heterodimer E2/E13 (Yin et al. 2009). The N-terminal domain, responsible for dimerization, contains four zinc finger motifs required for activation of the NFκB and JNK pathways (Chung et al. 2007). The C-terminal region of TRAF6 is responsible for interaction with the TNFα-like receptors including EDAR (Chung et al. 2007; Yin et al. 2009), and does not contribute to the activation of the NFκB pathway (Megas et al. 2011).

The interaction of EDARADD with TRAF6 recruits TAK1-binding proteins, TAB1 and TAB2 (Morlon et al. 2005). TAB1 and TAB2 activate the TAK1 (TGFβ–activated kinase 1) multisubunit complex composed of two kinases, IKK1/α and IKK2/β, which phosphorylate κB inhibitors α and β respectively, (DiDonato et al. 1997) as well as a structural component IKKγ (NEMO) (Deng et al. 2000). TAK1 probably contains other subunits such as Cdc37 and hsp90 (Chen 2005; Israel 2010). NEMO, which is composed of two coiled coil (CC) domains, interacts with the IKK1/α, IKK2/β through the N-terminal end of the CC1 domain, comprising a zinc finger motif (Chung et al. 2007), whereas the CC2 domain containing a leucine zipper is required for oligomerization of the complex (Agou et al. 2004). NEMO itself exhibits no catalytic properties and does not activate NFκB in response to TNFα, interleukin-1b, or lipopolysaccharide (Yamaoka et al. 1998).

Polyubiquitination of TRAF6 promotes activation of the TAB1/TAK1/TAB2 complex through several phosphorylation events involving various kinases, including a serine-threonine kinase IRAK (IL-1 receptor-associated kinase), an accessory protein recruited by interleukin-1 receptor and interacting with TRAF6 (Cao et al. 1996). It has been postulated that IRAK might play a role of an activator of EDAR signaling (Liu et al. 2008).

Another protein, CYLD which deubiqitinates TRAF6 (Jono et al. 2004; Kovalenko et al. 2003; Yoshida et al. 2005), and is negatively regulated by specific TNF receptors (Trompouki et al. 2003), might play a role of a silencer of EDAR signaling, since mutations in *CYLD* predispose not only to skin tumors (cylidromatosis) (Saggar et al. 2008), but also to the development of tumors of eccrine sweat glands and hair follicles (Brummelkamp et al. 2003; Trompouki et al. 2003).

Activation of the TAK1 complex leads to phosphorylation of IκBα, and IκBβ triggering their polyubiquitination and subsequent degradation in proteasomes, which determines the translocation of NFκB to the nucleus (Doffinger et al. 2001).

Depending on the activating signal and the cell type, two NFκB pathways, the canonical (depending on IKKβ and NEMO) and the non-canonical one (depending exclusively on IKKα) have been distinguished (Israel 2010).

**Fig. 1:**
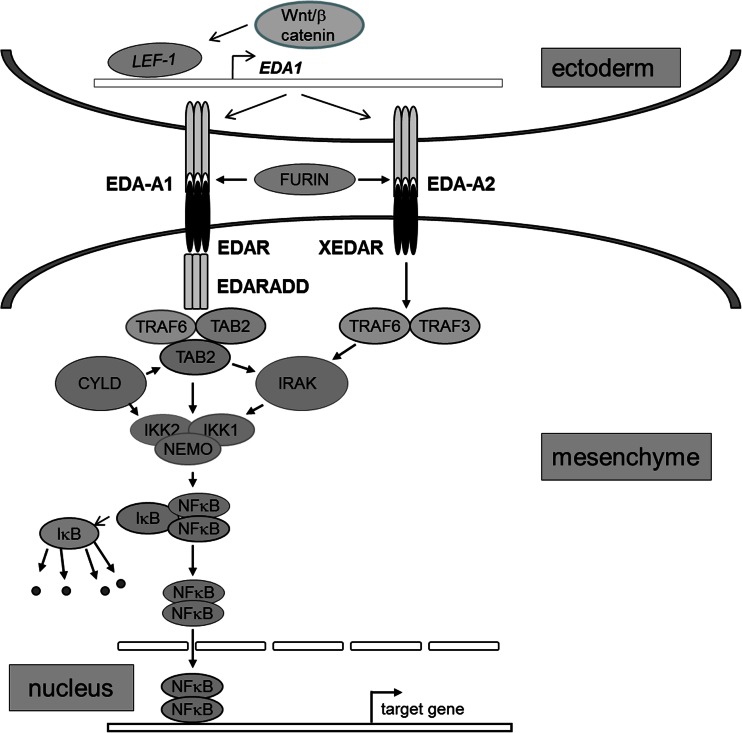
TNFα-related signal transduction pathway participating in the differentiation of skin appendages. Only EDA-A1, EDA1R and EDARADD are specific for the pathway. *ED1* is expressed in interfollicular cells. Its transcription is controlled by the Wnt/β-catenin pathway and requires LEF-1. The membrane-bound protein product of *ED1* is cleaved by furin-like proteases to obtain soluble ligands. EDA-A1 binds to EDAR which recruits EDARADD. Both are expressed in follicular cells. EDA-A2 binds to XEDAR, which does not require a protein adaptor to react with TRAF proteins. The remaining components are used by other signaling pathways that converge on NFκB. NFκB stimulates transcription of target genes, and their protein product interacts with several signaling pathways including Shh and BMP. Mutations of *ED1* cause X-linked HED, mutations of *EDA1R* or of *EDARADD* cause autosomal (either dominant or recessive) forms of HED. Mutations in *NEMO* cause X-linked HED associated with immunodeficiency, osteopetrosis, and lymphedema. For details see text

NFκB is a dimer composed of two out of five Rel-homology proteins (Perkins 2007). It remains bound to IκB and sequestered in the cytoplasm. After translocation to the nucleus, NFκB stimulates transcription of target genes (Smahi et al. 2002). The activation of NFκB is shown in Fig. 1.

Although in mice, deficient in a NFκB gene, no symptoms similar to the deficiency of Eda-A1 have been observed (Gugasyan et al. 2004), the phenotype of transgenic mice expressing a degradation-resistant IκB is very similar to that of Tabby mice suggesting that NFκB is essential for differentiation of skin appendages ( Schmidt-Ullrich et al. 2001). Moreover, it has been demonstrated that in the developing skin appendages, the Eda-A1-dependent expression of transgenic reporter constructs is required for the action of NFκB (Pispa et al. 2008; Schmidt-Ullrich et al. 2001), confirming earlier reports on NFκB involvement in the differentiation of skin appendages.

Thus EDA-A1, through EDAR and a number of signal transducing proteins, ultimately activates NFκB to stimulate transcription of the genes whose protein products are involved in the initiation, formation, and differentiation of skin appendages.

**Fig. 2:**
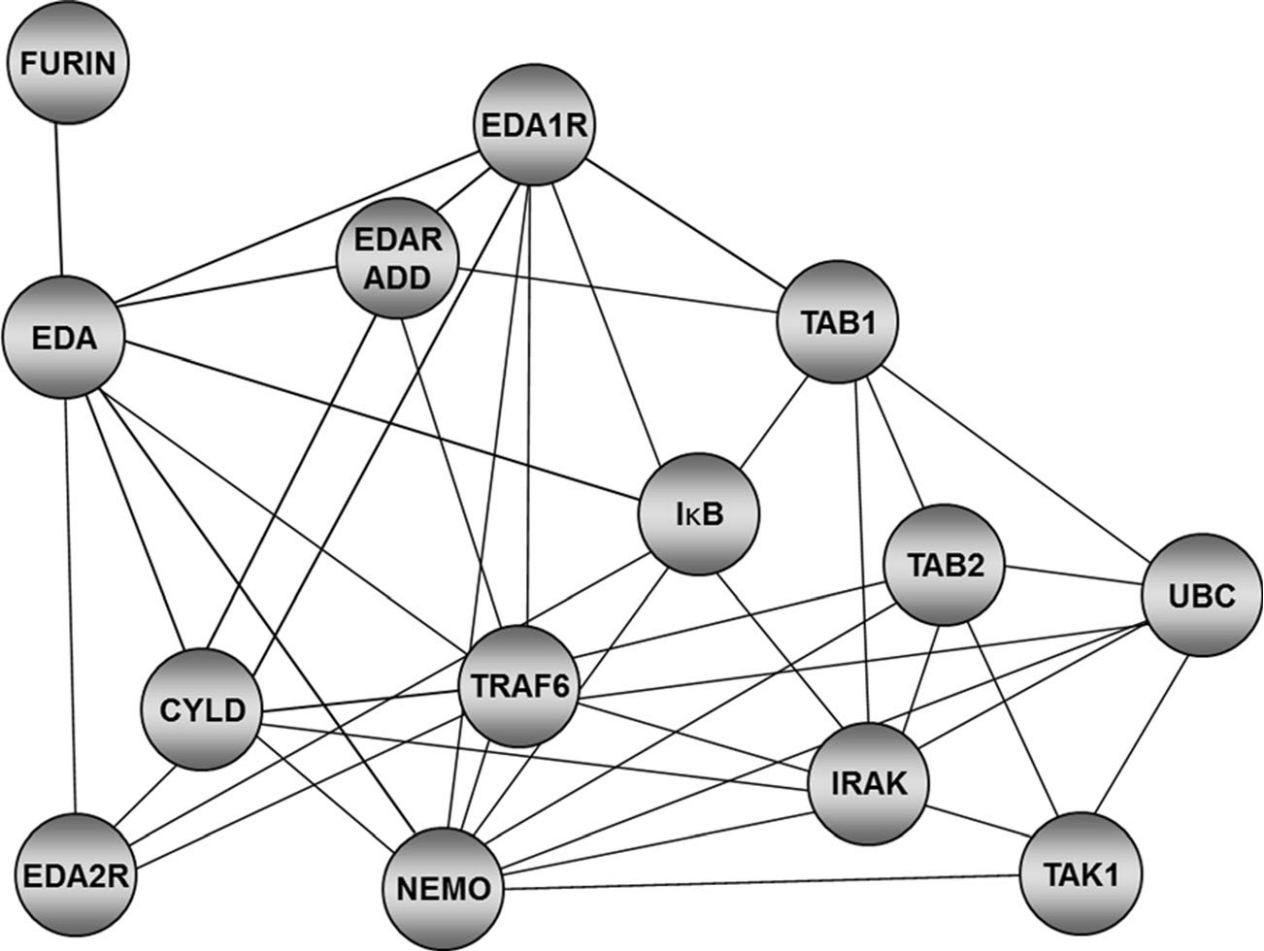
Interaction of proteins involved in signal transduction via the TNFα-related signal transduction pathway (based on http://string-db.org, simplified). EDA, two isoforms of ectodysplasin A; EDA-A1 isoform, binds to EDA-A1 receptor (EDAR); EDA-A2 isoform, binds to EDA-A2 receptor (XEDAR); EDARADD adapter protein, interacts with EDAR; CYLD, deubiqitinates TRAF6; FURIN, endopeptidase recognizing the RX(K/R)R sequence in EDA precursor; IκB, inhibitor NFκB; IRAK, indirect NFκB activator; NEMO, regulatory subunit of the IKK complex; TAK1, TGF-beta activated kinase 1; TAB1, activated kinase 1 binding protein; TAB2, activated kinase 2 binding protein; TRAF6, ubiquitin ligase; CYLD, deubiqitinates TRAF6; UBC, ubiquitin C

The complexity of the interactions between the constituents of signal transduction pathways initiated by EDA-1 and EDA-2 is illustrated by Fig. 2. Although the role of EDAR signaling in the processes of differentiation of skin appendages has been relatively well defined, the role of XEDAR signaling in these processes is not fully understood.

## Consequences of mutations of the genes encoding components of the TNFα-related signaling pathway

The vast majority of HED cases harbor mutations in *EDA*. Hemizygous males present the “classical” HED phenotype, but in the heterozygous female, expression of the symptoms varies considerably due to different levels of inactivation of the X-chromosome (Bartstra et al. 1994; Cambiaghi et al. 2000).

To date, more than 200 mutations of *EDA1* have been described. Most are point mutations and about 30 % constitute insertions or deletions, some leading to a frame shift and as a consequence, truncation of the protein product of the gene. About 20 % are mutations resulting from aberrant splicing (Table 1).

So far, over 40 mutations in *EDAR* have been reported. They account for about one-quarter of non-ED1-related HED (Chassaing et al. 2006). Out of these, about 60 % are point mutations, 25 % are insertions or deletions, and over 4 % are attributable to splicing defects. In homozygotes, the patients’ phenotype is similar to the “classical” one, but in the heterozygous carriers expression of the symptoms may be subtle or even negligible.

Only a few mutations in *EDARADD* (mostly point mutations and a single deletion) have been described (Chassaing et al. 2010). In homozygotes, the phenotype of the patients resembles the “classical” form of HED, while the phenotype of heterozygous carriers seems nearly normal, and upon careful inspection only minor abnormalities in the structure and function of skin appendages can be evidenced (Bal et al. 2007; Headon et al. 2001).

Over 90 mutations in *NEMO* have been reported so far. Nearly 50 % are point mutations, about 40 % are insertions or deletions and less than 10 % can be attributed to defects in splicing. These mutations, in addition to the defects typical of HED, produce immunodeficiency and *incontinentia pigmenti*.

Although symptoms typical of HED were described in Traf6 null mice (Naito et al. 2002), to date no mutations in *Traf6* were reported in mice, and until recently it was not known whether mutations of its human equivalent, *TRAF6* cause HED.

We reported the first mutation of *TRAF6* in a female patient displaying mild symptoms typical of HED (Wisniewski and Trzeciak 2012a). Sequencing analysis of one DNA strand in this patient revealed a deletion of eight nucleotides (c.1074-1081delCAACTTTG) in the 5’ fragment of the last exon of *TRAF6*, while no deletion was detected in the other DNA strand, indicating a heterozygous mutation. This deletion causes a shift of the reading frame and as a consequence, premature termination of translation 15 amino acids downstream of the mutation. This results in a truncated TRAF6 devoid of a major part of the C-terminal region that is responsible for interacting with EDAR. Because no mutations were found in *EDA*, *EDAR*, and *EDARADD*, and there were no signs of immunodeficiency and *incontinentia pigmenti*, which might suggest a mutation in *NEMO*, we postulated that this mutation was causative (Wisniewski and Trzeciak 2012a). Functional assays, involving coimmunoprecipitation studies and pull down assay, have clearly shown that the mutant TRAF6 protein is capable of forming a complex with TAK1 and TAB2, but it has completely lost the affinity to EDARADD (Fujikawa et al. 2013). These results confirmed our suggestion (Wisniewski and Trzeciak 2012a) that the novel mutation of *TRAF6* can cause symptoms of HED.

Because all genes mentioned above encode proteins of the same signal transduction pathway, it is not surprising that individuals having defects in one or more of these genes display a similar phenotype and exhibit similar symptoms, albeit of a different intensity of expression (Kobielak et al. 2001).

Until recently, no mutations were reported in the genes encoding other proteins involved in the interaction with TRAF6 and translocation of NFκB to the cell nucleus, except of the mutation located in the gene encoding IκBα which caused the autosomal dominant form of ectodermal dysplasia associated with T-cell deficiency (Courtois et al. 2003).

Substantial evidence indicates that XEDAR upon interaction with EDA-A2 induces caspase-dependent apoptosis in osteosarcoma cell lines (Chung et al. 2007; Sinha and Chaudhary 2004), while expression of a dominant negative XEDAR suppresses feather development in chickens (Drew et al. 2007). Moreover, in humans, a single nucleotide polymorphism of *XEDAR* was associated with male-pattern baldness (Prodi et al. 2008; Redler et al. 2012) and it was demonstrated that XEDAR, like EDAR, interacts with TRAF6 (Naito et al. 2002). Taken together, these findings indicate that XEDAR might participate in signal transduction from ectoderm to mesenchyme, and that mutations in *XEDAR* might disturb differentiation of skin appendages. However, no symptoms typical of ectodermal dysplasia were reported in *Xedar* knock-out mice (Newton et al. 2004) and until recently, no mutations in *XEDAR* were found in patients with symptoms typical of HED, suggesting that XEDAR is not involved in the differentiation of skin appendages.

However, we have reported a new mutation (c.252delG) of *XEDAR* in a patient with symptoms typical of HED, in whom no mutations in *EDA*, *EDA1R*, *EDARADD*, and *TRAF6* have been detected (Wisniewski and Trzeciak 2012b). This novel mutation leads to a frameshift and as a consequence to a deletion of the entire intracellular and transmembrane domains of XEDAR.

Although the Eda-A2/Xedar pathway has not been associated with the mouse variant of HED, it has been demonstrated that Xedar activates the NF-κB pathway in a ligand-dependent fashion with the mediation of Traf6 and the kinases of the IKK complex (Naito et al. 2002). Moreover, it has been shown that Traf6 binds strongly to the intracellular domain of Xedar, and that Traf6-deficient mice display symptoms typical of the mouse variant of HED (Naito et al. 2002). These observations suggest that the protein products of both *Edar* and *Xedar* can interact with TRAF6 and function in the NFκB signaling.

Based on our results (Wisniewski and Trzeciak 2012b), it could be reasoned that the deletion of an entire TRAF6-interacting domain of XEDAR makes activation of the NFκB pathway impossible.

Thus under normal conditions the protein product of *XEDAR* might participate in the differentiation of skin appendages and this mutation might be responsible for the symptoms typical of HED. This will be the first mutation in *XEDAR* associated with symptoms of HED. However, to confirm our findings (Wisniewski and Trzeciak 2012b) that the (c.252delG) mutation is causative a functional assay is required.

## Chromosomal localization and the mode of inheritance of mutations of genes responsible for HED

*EDA* is located on the X chromosome (Xq12-q13) and mutations of this gene are therefore transmitted by heterozygous mothers to their offspring. This is the reason why the symptoms of HED (ECTD, 300451) are much more pronounced in the hemizygous males than in the heterozygous females who are carriers of these mutations (Table 1).

*EDAR* is situated on chromosome 2q12.3 (Monreal et al. 1999). Mutations in this gene are therefore inherited in either autosomal dominant (ECTD10A, phenotype MIM 129490) (Fujikawa et al. 2013; Fujimoto et al. 2008; Monreal et al. 1999; van der Hout et al. 2008) or autosomal recessive way (ECTD10B, phenotype MIM 224900) (Chassaing et al. 2006; Lind et al. 2006; Megarbane et al. 2008; Naeem et al. 2005; Shimomura et al. 2004).

*EDARADD* localization (1q42.3) indicates that mutations of this gene can be inherited in either an autosomal dominant or recessive way (Bal et al. 2007). Similarly, deficiency of EDAR causes either autosomal dominant (ECTD11A, phenotype MIM 614940) or autosomal recessive (ECTD11B, phenotype MIM 614941) forms (Bal et al. 2007; Headon et al. 2001; Munoz et al. 1997).

*NEMO* like *EDA* and *XEDAR* is located on the X chromosome (Xq28) and mutations of this gene are usually transmitted by heterozygous mothers, but “typical” symptoms of HED are seen only in hemizygous males. In heterozygous females the symptoms are mild or negligible. The symptoms of HED due to in frame mutations in *NEMO* (OMIM 308300) are most often accompanied by immunodeficiency and *incontinentia pigmenti* (EDA-ID). When mutations lead to truncation of a protein product of the gene, immunodeficiency is accompanied by osteopetrosis and lymphedema (OL-EDA-ID). In most cases a severe genodermatosis is associated with hypodontia, peg-shaped or malformed teeth and in some patients ophtalmological and neurological problems (Chassaing et al. 2010).

*TRAF6* is located on chromosome 11p12. The first mutation reported in *TRAF6* (Wisniewski and Trzeciak 2012a) originated *de novo*. Theoretically, the inheritance pattern of HED due to mutations in *TRAF6*, should be autosomal dominant, as in our patient, since only mild symptoms of HED were detected in his heterozygous mother.

The novel mutation found in *XEDAR*, which is located on the X chromosome (20p1), similar to mutations in *EDAR* and *NEMO*, was transmitted by a heterozygous mother to her son (Wisniewski and Trzeciak 2012b).

## Clinical symptoms and prosthetic treatment of hypohidrotic ectodermal dysplasia

The main clinical symptoms characteristic of HED include reduced ability to sweat (hypohidrosis), the lack of several or more teeth (hypodontia), and sparse hair (hypotrichosis).

**Fig. 3:**
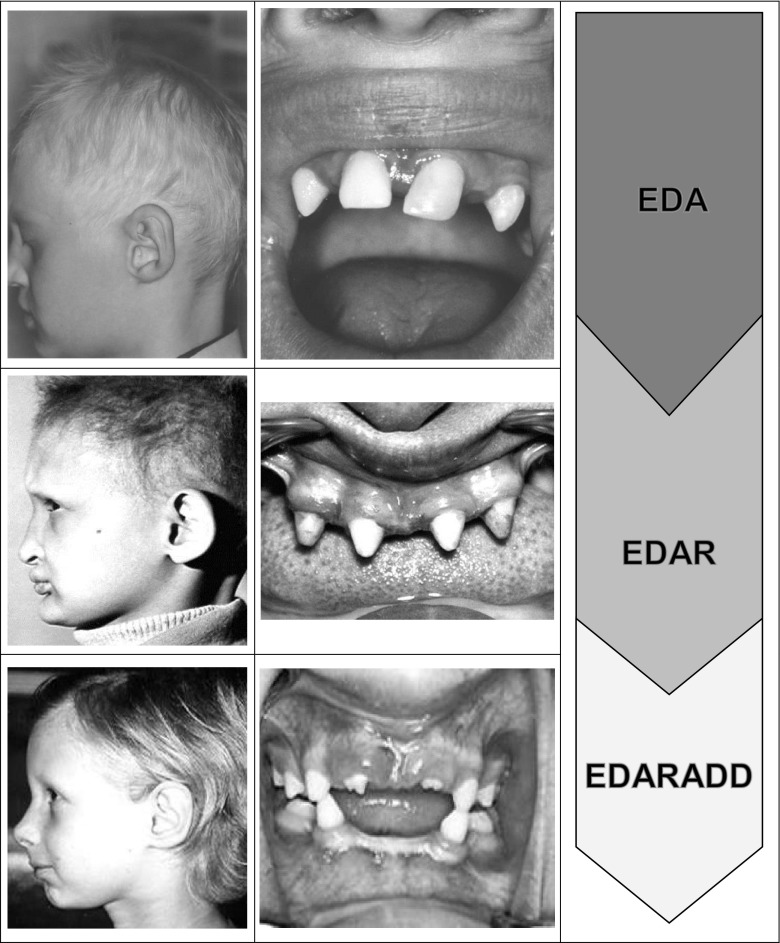
Phenotype of patients with HED caused by mutations of TNF-related signaling pathway. (Reproduced with permission of the editor from: Wiśniewski S.A., Trzeciak W.H., *Homines hominibus* 6: 21–28, 2010 (a) Side view of the patient; (b) dentition; (c) mutated gene

Reduction in the ability to sweat causes overheating and this in turn results in about 30 % mortality rate in early childhood if the disease is not properly diagnosed and treated (Clarke et al. 1987). Consequently, due to disturbed thermal perception, the patients suffer from overheating (hyperthermia), particularly in the Summer. Dentition abnormalities include incorrect numbers and shape of teeth (Fig. 3) and leads to impaired mastication and speech defects as well as an esthetic discomfort. Defects in the number of teeth are very common. Third molars (wisdom teeth) do not develop in 25 % of the world’s population. The number of teeth range from over a dozen (hypodontia) through several (oligodontia) to the lack of all permanent teeth (anodontia) or even the lack of both permanent and decidual teeth (aplasia) (De Coster et al. 2009; Nieminen 2009).

The teeth are frequently conical, bulbous or taurodontic and widely spaced, without any points of contact. The compromised enamel is prone to caries and mechanical damage. Tooth eruption can be obstructed and the number of teeth can be significantly reduced. Some teeth fail to emerge in the oral cavity.

Atrophic inflammation of the mucosa of the oral cavity and the throat, a hoarse voice, and swallowing difficulties are also signs of HED.

The patients’ hair is thin, sparse, fragile and light-colored. The skin is thin, pale, very delicate and is dry and exfoliating with insufficient pigmentation, except for the region around the eyes and mouth where it is wrinkled and hyperpigmented, giving a prematurely aged appearance (Mikkola 2009; Wisniewski et al. 2002). The skin is vulnerable to infection, irritation, and mechanical trauma and this can be attributed to the dysfunction of the dermal glands and the presence of a thin fatty layer. The skin on the palms and soles exhibits characteristic dermatogliphic patterns. The nails can be poorly or abnormally formed and discolored. The ocular surface abnormalities (corneal lesions and inflammation), caused by a lack of Meibomian’s glands, have also been reported (Cui et al. 2005; Kaercher 2004).

All of these manifestations are often accompanied by impaired vision and hearing as well as frequent infections of the respiratory tract (Dietz et al. 2013) that can be attributed to dry airways and mucous membranes.

Some patients display facial dysmorphism and other abnormalities including a prominent forehead, depressed nasal bridge, thick lips, and/or a pointed chin.

## Treatment of patients with hypohidrotic ectodermal dysplasia

Although HED gene therapy has been attempted in mice (Gaide and Schneider 2003) and dogs (Casal et al. 1997, 2007) to date no appropriate protocol for gene therapy in humans has been reported.

There is not much we can do for the patient in terms of treatment. Treatment involves avoidance of overheating and application of creams that protect the dry and delicate skin from possible damage. Tooth abnormalities can be corrected by prosthetic treatment which needs to be highly individualized due to variation in the degree of hypo- or oligodontia.

**Fig. 4:**
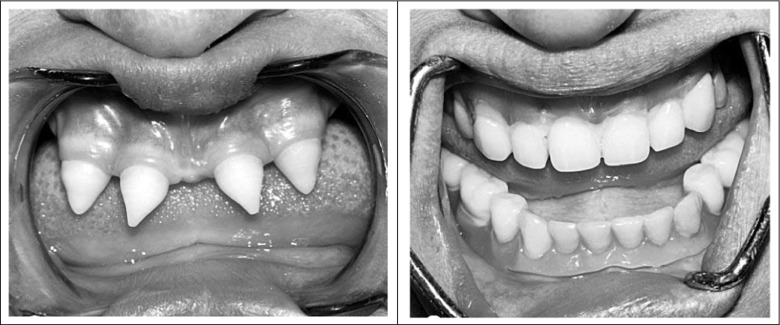
The effects of prosthetic treatment of one of our patients with HED caused by mutation of *EDAR* (**a**) before the treatment. Note four teeth of conical shape in mandible and none in maxilla. (**b**) after the prosthetic treatment

Removable dentures of a varying plate size can be used in the case of persons aged up to 18 years. Generally, partially removable prostheses with a small number of retention clasps are applied in order to ensure the proper development of the prosthetic base bone structure. Effective anchoring of such dentures requires preparing retention sites with the use of composite materials on existing, abnormally formed teeth, which are often icicle-shaped. Adaptation of the removable prosthesis is necessitated by structural bone changes in the base, coupled with the shape of the few remaining teeth in the dental arch, and involves relining or replacement of the restoration (Fig. 4). It is very important to prepare the acrylic mucosal part of the complete or partial denture in the proper way. The use of thin tin foil in the dental laboratory protects the dry and delicate oral mucosa from the traumatic impact of the plate. Emphasis should also be placed on systematic check-ups of the prosthetic base, denture corrections, and instructing the patient to apply specific hygienic procedures.

In the case of anodontia, removable prostheses should be applied. If the patient is under 18 years of age, the prostheses should be periodically replaced by new, larger ones, to ensure the proper development of the bone structure of the prosthetic base.

## Concluding remarks

Within the last years, considerable progress has been made in better understanding the process of signal transduction from ectoderm to mesenchyme. This signal is responsible for the initiation, formation, and differentiation of skin appendages. The structure of the genes encoding the components of the TNFα-related signaling pathway has been resolved and the functions of the protein products of these genes precisely characterized. Functional assays have been developed for nearly all of these proteins. A number of new mutations of the genes encoding components of the TNFα-related signaling pathway have been reported. However, these new discoveries have rarely been supported by functional assays and this has cast doubt whether all of the reported mutations are causative. This applies mainly to the missense mutations which usually do not change the structure of a protein product of the gene to a large extent. Nucleotide insertion or deletion within the sequence of a gene does not necessarily require a functional assay because such mutations usually cause a shift in the reading frame and as a result, truncation of the protein, which may lack some functionally important domain(s) which interact with other proteins of the TNFα-related signaling pathway.

Moreover, the normal structure of the gene is not sufficient to guarantee that the protein product of this gene is synthesized, because the gene might not be expressed. In the case where mutations in the genes encoding proteins involved in the TNFα-related signaling pathway were not detected, it should therefore be proved that the genes were expressed. Therefore, further investigations on the mode of transcriptional regulation of the genes encoding components of the TNFα-related signaling pathway are still required.
